# Dietary Fermented Mulberry Leaf Triggers Dose-Dependent Changes in Growth, Antioxidant Immune Status and Intestinal Microecology of Amur Sturgeon (*Acipenser schrenckii*)

**DOI:** 10.3390/ani16182918

**Published:** 2026-09-16

**Authors:** Nan Zhang, Wanwan Zhu, Xuekai Wang, Yi Xiong, Sasa Zuo, Fuyu Yang, Yongsheng Wang

**Affiliations:** 1 Department of Grassland Science, College of Animal Science, Guizhou University, Guiyang 550000, China; zn2580042898@126.com (N.Z.); wan850793534@126.com (W.Z.); 2 Beijing Tianyuanyugang Farm Co., Ltd. (Beijing Amur Sturgeon Elite Breeding Base), Beijing 101400, China; 3 College of Grassland Science and Technology, China Agricultural University, Beijing 100193, China; xkwang2016@163.com; 4 Frontier Technology Research Institute of China Agricultural University in Shenzhen, Shenzhen 518000, China; xiongleslie@126.com; 5 College of Engineering, China Agricultural University, Beijing 100193, China; zss@cau.edu.cn; 6 Shenzhen Branch, Guangdong Laboratory of Lingnan Modern Agriculture, Key Laboratory of Livestock and Poultry Multi-Omics of MARA, Agricultural Genomics Institute at Shenzhen, Chinese Academy of Agricultural Sciences, Shenzhen 518000, China

**Keywords:** *Acipenser schrenckii* (Amur sturgeon), dose-dependent responses, fermented mulberry leaf, growth performance, intestinal microecology, metabolomics

## Abstract

Mulberry leaf is a promising sustainable plant-based feed resource; however, high dietary inclusion of raw mulberry leaf impairs growth in carnivorous fish. Fermentation degrades anti-nutritional components and enhances nutrient availability, yet the dose-dependent effects and underlying mechanisms of fermented mulberry leaf (FML) in *Acipenser schrenckii* remain unclear. This study assessed graded dietary FML inclusion (0%, 2%, 4%, and 6%) as a partial replacement for fishmeal in Amur sturgeon, measuring growth performance, intestinal morphology, serum biochemistry, target gene expression, gut microbiota, and metabolome. Moderate FML maintained acceptable growth, increased villus height, activated the Keap1–Nrf2 antioxidant pathway, and raised serum IgM and C3 levels without provoking severe intestinal inflammation. Intestinal microecology underwent prominent dose-dependent remodeling: low-dose FML enriched *Lysinibacillus* and *Clostridium_T*, while moderate-to-high FML increased the abundance of beneficial Bacillales taxa that modulated amino acid and energy-associated metabolic pathways. Quadratic regression estimated an optimal FML dosage of 3.66–4.62%. The 4% FML group achieved balanced improvements in growth and intestinal health within this optimal range. Collectively, FML represents a promising sustainable feed ingredient for sturgeon aquaculture, providing new references for applying mulberry by-products in carnivorous fish diets.

## Simple Summary

Mulberry leaves are a promising plant-based feed material for aquaculture, but raw mulberry leaves contain anti-nutritional factors that limit their use in carnivorous fish. Fermentation can break down these harmful components and improve nutrient availability. In this study, we evaluated the effects of adding graded levels (0%, 2%, 4%, and 6%) of fermented mulberry leaf (FML) to the diets of Amur sturgeon (*Acipenser schrenckii*) as a partial replacement for fishmeal. Our results showed that moderate FML inclusion (2–4%) maintained growth performance comparable to the control group while improving intestinal structure, enhancing antioxidant capacity, and boosting immune responses without causing intestinal inflammation. High-dose FML (6%) resulted in reduced growth despite favorable effects on gut microbiota. The optimal FML dosage was estimated at 3.66–4.62% based on mathematical modeling. In conclusion, adding 4% fermented mulberry leaf to sturgeon feed offers a balanced improvement in both growth and health, supporting its potential as a sustainable ingredient for carnivorous fish diets. These findings provide practical guidance for the use of mulberry by-products in aquaculture.

## 1. Introduction

*Acipenser schrenckii* is a rare and economically valuable sturgeon species endemic to China. As an ancient Jurassic vertebrate, the sturgeon is regarded as a genetic “living fossil” with high scientific research value [1]. Sturgeon meat provides nutrients beneficial to human metabolism and cardiovascular health, while sturgeon caviar is globally recognized as a high-grade luxury aquatic product [2]. Driven by its prominent economic and research value, commercial sturgeon aquaculture has developed rapidly worldwide [3,4]. China dominates the global sturgeon aquaculture industry, achieving a total sturgeon output of 149,400 tons and a caviar export volume of 275.83 tons in 2023, ranking first worldwide in farmed sturgeon caviar production [5]. As obligate carnivorous fish, sturgeons rely heavily on high-fishmeal diets. However, the increasing scarcity and rising cost of fishmeal have promoted the substitution of plant-based protein sources in modern aquafeeds. Nevertheless, high-proportion plant protein replacement frequently disrupts intestinal microbial homeostasis, impairs intestinal barrier function, reduces antioxidant and immune capacities, and increases disease susceptibility in aquatic animals [6,7].

The fish intestine is a critical dual-functional organ responsible for nutrient digestion and absorption and acts as a primary physical and immune barrier against invasive pathogens and harmful toxins. The intestinal immune defense system mainly depends on gut-associated lymphoid tissues (GALT) in the intestinal epithelium and lamina propria, together with secreted cytokines and immunoglobulins [8]. The intestinal microbiota is a core regulator of fish growth, nutrient metabolism, immune response, and disease resistance. The relative proportions of dominant phyla, including Fusobacteria, Firmicutes, Bacteroidetes, and Proteobacteria, are widely used as key biomarkers to evaluate diet-induced variations in intestinal microbial structure and ecological function [9,10].

Antibiotic overuse in aquaculture has accelerated the prevalence and transmission of antibiotic resistance, posing severe threats to global public health, food safety, and sustainable aquaculture development [11,12]. Therefore, screening efficient, safe, and sustainable alternative feed ingredients to partially replace fishmeal, improve intestinal health, and reduce antibiotic dependence is essential for eco-friendly sturgeon aquaculture.

Mulberry leaf is a typical medicinal and edible plant resource with outstanding application potential in animal feed. China produces over 15 million tons of mulberry leaves annually, representing abundant and low-cost biomass resources [13]. Mulberry leaves contain up to 36% crude protein, together with rich amino acids, minerals, and dietary fiber, exhibiting favorable nutritional characteristics for feed substitution [14]. In addition, mulberry leaves are rich in bioactive substances, including polysaccharides, polyphenols, and alkaloids, which possess antioxidant, anti-inflammatory, and hypoglycemic biological activities [15]. Multiple studies have verified that dietary mulberry leaf supplementation improves growth performance, antioxidant capacity, and immune status in livestock and poultry [16,17]. However, raw mulberry leaves contain tannins and crude fiber, which restrict their application in aquatic feeds. Microbial fermentation can effectively degrade anti-nutritional factors and crude fiber, significantly improving the nutritional value and digestibility of mulberry leaves [18,19]. Moreover, mulberry leaf bioactive compounds have been confirmed to alleviate intestinal microbial dysbiosis and restore intestinal microecological balance [20], indicating great potential for regulating fish intestinal health.

Currently, few studies focus on the application of fermented mulberry leaves (FMLs) in sturgeon aquaculture, and the regulatory mechanisms underlying FML-modulated growth, immunity, and intestinal microecology in *A. schrenckii* remain unclear. Accordingly, this study incorporated graded levels of FML into sturgeon diets as a partial replacement for fishmeal and comprehensively evaluated its effects on growth performance, antioxidant capacity, immune response, intestinal microbiota, and metabolic characteristics via multi-omics analysis. This study aims to provide a theoretical basis and practical reference for the application of fermented mulberry leaf resources in sturgeon culture and to promote the sustainable development of eco-friendly aquaculture.

## 2. Materials and Methods

### 2.1. Animal Ethics

All experimental procedures were carried out following the Chinese Laboratory Guidelines for the Ethical Review of Animal Welfare (National Standard, GB/T 35892-2018 [21]), and the experiments were approved by the Inspection Form for the Guizhou University Experimental Animal Ethics (approval No. EAE-GZU-20240E072, approval date 31 December 2024).

### 2.2. Experimental Diets

Four isonitrogenous and isoenergetic experimental diets were formulated. The basal diet contained 55% fishmeal and served as the control group (CK). In the three experimental diets, fishmeal was partially replaced by 2%, 4%, and 6% fermented mulberry leaf (FML), designated FML2, FML4, and FML6, respectively. All diets were formulated to be isonitrogenous and isoenergetic by adjusting other protein and energy sources accordingly. Dried raw mulberry leaves were purchased from Shichao Fruit and Vegetable Planting Farmers Professional Cooperative (Shangqiu, China) and fully pulverized before feed preparation.

The mulberry leaf fermentation protocol was adopted in accordance with Zuo et al. [22]. Briefly, raw mulberry leaves were sterilized at 121 °C for 20 min. After cooling to room temperature (RT), the substrates were inoculated with a mixed spore suspension of *Aspergillus oryzae* (GenBank accession number: KU320673) and *Saccharomyces cerevisiae* (GenBank accession number: KM005256). Subsequently, the mixture was incubated in a biochemical incubator at 28 °C for 3 days for fermentation. After fermentation, proximate composition (dry matter, crude protein, crude lipid, crude ash, and crude fiber) and tannin concentrations of raw mulberry leaves and fermented mulberry leaves were measured, and all data were summarized in Table S1. The prepared FML was incorporated into the corresponding experimental diets accordingly.

Crystalline amino acids (CAA; L-Lys-HCl, DL-Met, L-Thr), fish oil, and monocalcium phosphate were supplemented to balance essential amino acids (EAA), essential fatty acids, and available phosphorus, respectively. The supplementation strategy referred to the EAA requirements of *Acipenser transmontanus* reported by Ng and Hung [23] and a previous study conducted by Wei [24]. The formulation and proximate nutritional composition of all experimental diets are presented in Table S2, while dietary amino acid profiles are provided in Table S3. All diets were extruded into 3 mm sinking pellets using an EXT50A twin screw extruder (Yanggong Machinery Co., Ltd., Beijing, China). The temperatures of the feeding zone, compression zone, and metering zone were set at 92 °C, 121 °C, and 87 °C, respectively. Pellets were air-dried at room temperature and preserved at −20 °C until feeding trials.

Proximate nutritional analyses of experimental diets followed official methods issued by AOAC INTERNATIONAL [25]. Dry matter content was determined by oven drying to constant weight at 105 °C. Crude protein was quantified using the Kjeldahl method. Crude lipid was measured via Soxhlet extraction. Crude ash content was obtained after combustion in a muffle furnace. Crude fiber was detected based on the filter bag method described in the Chinese national standard GB/T 6434-2022 [26]. Full amino acid profiling was completed by Evonik Industries (Beijing, China).

### 2.3. Experimental Fish and Feeding

A total of 1000 *Acipenser schrenckii* juveniles were purchased from Beijing Amur Sturgeon Elite Breeding Base (Beijing, China). Before the formal trial, 320 fish with an initial body weight (Wo) of 199.08 ± 1.29 g were acclimated to experimental culture conditions for 4 weeks and fasted for 24 h. Fish were randomly allocated into 16 net cages (1 m × 1 m × 1.5 m), with 20 fish per cage. Four net cages were set as four biological replicates for each dietary treatment. Fish were fed twice daily for 10 weeks. Water quality parameters were maintained throughout the experiment: temperature, 20 ± 2 °C; pH 7.5–8.5; dissolved oxygen > 7.0 mg/L.

### 2.4. Sample and Data Collection

Feed intake, fish mortality, and body weight were recorded regularly during the feeding period. At the end of the trial, all fish were fasted for 24 h before sampling. Each dietary treatment consisted of four replicate cages, and three fish were randomly selected from each cage, yielding a total of 12 fish per group. These 12 fish were anesthetized using MS-222 (100 mg/L) and used for subsequent parallel analyses. Final body weight, body length, liver weight, and visceral weight were measured to calculate growth-related indicators. Blood was collected from the caudal vein. After standing at room temperature for 30 min, blood samples were centrifuged at 4 °C to harvest serum for biochemical detection. Foregut tissues were snap-frozen in liquid nitrogen and preserved at −80 °C to detect intestinal digestive enzyme activities. A 2 cm foregut segment was fixed in 4% paraformaldehyde for histological observation. Approximately 0.5 g of midgut and hindgut tissues were immersed in RNALater (Beyotime, Shanghai, China), incubated at 4 °C overnight, and stored at −80 °C for RNA extraction. For untargeted metabolomics and intestinal microbiota analysis, six samples per group were randomly selected from the 12-fish pool, with all four cages represented (at least one fish per cage). For serum biochemical indices, intestinal digestive enzyme activities, and qPCR analyses, samples were derived from three independent cages (one fish per cage). All assays were conducted in parallel using independent samples.

### 2.5. Growth Performance

Calculations of weight gain rate (WGR), specific growth rate (SGR), feed conversion ratio (FCR), feeding rate (FR), survival rate (SR), condition factor (CF), hepatosomatic index (HSI), and viscerosomatic index (VSI) were conducted with the formulas listed below. Wt is the terminal body weight; Wo is the original body weight, and the same applies below.

WGR (%) = 100 × (Wt − Wo)/Wo.

SGR (%/day) = 100 × [(ln (Wt) − ln (Wo)]/days of feeding trial.

FCR = feed intake/(Wt − Wo + weight of dead fish).

FR (%) = 100 × total dry matter intake/[(Wt + Wo)/2 × days of feeding trial].

SR (%) = 100 × terminal quantity/original quantity.

CF (g/cm^3^) = 100 × body weight/(body length)^3^.

HSI (%) =100 × hepatic weight/body weight.

VSI (%) =100 × visceral weight/body weight.

### 2.6. Biochemistry Indices

Serum biochemical parameters were determined using commercial kits. Immunoglobulin M (IgM, Cat. No. IG7437), complement 3 (C3, Cat. No. C37392), and complement 4 (C4, Cat. No. C47402) were measured with ELISA kits (Beijing Leadman Biochemical Co., Ltd., Beijing, China). Total antioxidant capacity (T-AOC, Cat. No. A015-2), superoxide dismutase (SOD, Cat. No. A001-1), malondialdehyde (MDA, Cat. No. A003-1), and intestinal amylase (AMS, Cat. No. C016-1-1), lipase (LPS, Cat. No. A054-2-1), and trypsin (Cat. No. A080-2-1) were quantified using corresponding kits (Nanjing Jiancheng Bioengineering Institute, Nanjing, China). All assays followed the manufacturers’ protocols.

### 2.7. Intestinal Histology

After fixation in 4% paraformaldehyde, intestinal tissues underwent dehydration, embedding, sectioning, and hematoxylin and eosin (HE) staining. For each treatment group, intestinal sections were prepared from 3 randomly selected fish. From each section, 5 villus heights and their corresponding 5 crypt depths were randomly selected and measured. Villus height and crypt depth were observed under a Nikon Eclipse E100 microscope (Nikon, Tokyo, Japan) and measured using Image-Pro Plus 6.0 software (Media Cybernetics, Rockville, MD, USA). The mean values for each fish were calculated and used for statistical comparisons.

### 2.8. Intestinal Microbiota Diversity Analysis

Intestinal microbiota profiling was performed by extracting total bacterial DNA from intestinal tissues and digesta, followed by PCR amplification of the V3–V4 hypervariable region of the 16S rRNA gene using primers 338F and 806R. DNA quality and concentration were assessed by agarose gel electrophoresis and NanoDrop 2000 spectrophotometry (Thermo Fisher Scientific, Waltham, MA, USA). Purified PCR products (Vazyme VAHTSTM DNA Clean Beads, Nanjing Vazyme Biotech Co., Ltd., Nanjing, China) were quantified with the Quant-iT PicoGreen dsDNA Assay Kit (Invitrogen, Waltham, MA, USA), pooled in equimolar amounts, and sequenced on an Illumina NovaSeq 6000 platform (2 × 250 bp paired-end; NovaSeq 6000 SP Reagent Kit, 500 cycles) at Shanghai Personal Biotechnology Co., Ltd., Shanghai, China.

Raw sequences were denoised, and ASVs were clustered using the DADA2 and Vsearch plugins within QIIME2. Microbial diversity was assessed by alpha diversity indices (Chao1, observed species, Shannon, Simpson) and visualized by non-metric multidimensional scaling (NMDS) based on Bray–Curtis distances. Community structure differences were evaluated using PERMDISP and Adonis. Differential taxa were identified by the Kruskal–Wallis H test and Wilcoxon rank sum test, with LEfSe (LDA > 2, *p* < 0.05) applied for biomarker selection.

### 2.9. Untargeted Metabolomics Analysis Based on LC-MS/MS

A total of 30 mg of intestinal tissue was homogenized twice (55 Hz, 60 s) in pre-cooled ultrapure water with steel beads. Samples were mixed with methanol–acetonitrile (1:1, *v*/*v*), ultrasonicated for 30 min, incubated at −20 °C for 45 min, and centrifuged at 12,000 rpm (4 °C, 20 min). The supernatant was vacuum-dried, reconstituted in 50% methanol containing 5 ppm 2-chlorophenylalanine, vortexed, and centrifuged. The liquid was filtered with a 0.22 μm membrane and transferred to sample vials.

Metabolite detection was performed on a Vanquish UHPLC system (VQ-COREISO-01, Thermo Fisher Scientific, USA) coupled with an Orbitrap Exploris 120 mass spectrometer (BRE725531, Thermo Fisher Scientific, USA) equipped with an electrospray ion source. The analytical workflow followed Chen et al. [27]. Metabolites were detected in positive (POS) and negative (NEG) ion modes. Orthogonal partial least squares discriminant analysis (OPLS-DA) distinguished metabolic profiles of the control and FML groups. Differential metabolites were screened using criteria of variable importance in projection (VIP) > 1, *p* < 0.05, and fold change (FC) > 1. Metabolites from the two ion modes were merged; redundant metabolites were removed by retaining compounds with higher VIP values.

Metabolic pathway enrichment was annotated against the Kyoto Encyclopedia of Genes and Genomes (KEGG) database with reference to metabolic pathways reported in *Acipenser ruthenus*, a closely related sturgeon species, because a well-annotated reference genome for *A. schrenckii* is currently unavailable. It should be noted that this cross-species pathway mapping is approximate and may not capture sturgeon species-specific metabolic routes. Fisher’s exact test identified significantly enriched pathways regulated by dietary FML inclusion.

### 2.10. Correlation Analysis of Microorganisms and Metabolites

Spearman rank correlation analysis was conducted between genus-level differential gut microbiota and differential metabolites. Correlation pairs with *p* < 0.05 and absolute correlation coefficient |corr| > 0.5 were retained. The top 20 microbes and metabolites ranked by correlation magnitude were selected to build clustered heatmaps for visualizing positive and negative correlations. In heatmaps, red represented significant positive correlations, blue–purple indicated significant negative correlations, and asterisks marked statistically significant correlations.

### 2.11. RNA Isolation, Reverse Transcription, and mRNA Level Analysis

Total RNA was extracted from intestinal tissue using the RNA EasyFast Kit (Tiangen Biochemical Technology Co., Beijing, China). Total RNA was treated with DNase I to eliminate genomic DNA contamination. RNA integrity was verified by 1% agarose gel electrophoresis, and RNA concentration and OD260/280 ratios were measured using a NanoDrop 2000 spectrophotometer (Thermo Fisher Scientific, Waltham, MA, USA), with OD260/280 ratios ranging from 1.80 to 2.00.

The reference gene *β-actin* (GenBank accession No. AY649619) and target gene primers for *A. schrenckii* were obtained from Wei [6] and are listed in Table S4. In the present study, the Ct values of *β-actin* across all samples were highly stable, ranging from 18.33 to 18.53 (mean ± SD: 18.41 ± 0.10, coefficient of variation = 0.54%), supporting its suitability as an internal reference gene under our experimental conditions. RT-qPCR was carried out on a qTOWER Iris instrument (Analytik Jena, Jena, Germany) with TB Green^®^ Premix Ex Taq™ II (Takara, Tokyo, Japan), using three biological replicates (one fish from each of three independent cages), with three technical replicates per sample. No-template controls (NTC) were included on each qPCR plate to monitor reagent contamination, and no amplification signal was detected in any NTC reaction. The two-step amplification program included initial denaturation at 95 °C for 30 s, followed by 40 cycles of 95 °C for 5 s and combined annealing and extension at 60 °C for 34 s. Relative gene expression was quantified using the 2^−ΔΔCt^ method.

### 2.12. Data Analysis

All statistical analyses were implemented in GraphPad Prism 10.6.0 (San Diego, CA, USA). Normality and homogeneity of variance were verified prior to parametric statistics. To properly address the hierarchical structure of the experimental design and avoid pseudo-replication, different analytical units were adopted according to the sampling strategy for each assay. For growth performance indicators, data were collected from all fish in each cage and averaged to obtain cage-level values; these were analyzed using one-way ANOVA with cage as the experimental unit (*n* = 4 per treatment). For serum biochemical indices, intestinal digestive enzyme activities, and RT-qPCR, samples were derived from three independent cages (one fish per cage); cage means were used as the statistical unit for one-way ANOVA (*n* = 3 per treatment). For 16S rRNA sequencing and untargeted metabolomics, six samples per treatment were selected from the 12-fish pool covering all four cages; cage identity was included as a random effect in the mixed-effects model (for metabolomics) and as a blocking factor in PERMANOVA (for microbiota) to account for inter-cage variation. This stratified analytical framework ensured that all statistical inferences were based on cage-level replication, thereby eliminating pseudo-replication at the individual fish level. One-way ANOVA was used for all phenotypic indicators across four dietary groups, with Tukey’s test for multiple comparisons; different superscript letters denoted significant differences. Two-way ANOVA was applied for RT-qPCR expression data.

One-way ANOVA model:

*Y_ij_* = *μ* + *T_i_* + *ε_ij_*

*Y_ij_* = observation of replicate *j* in treatment *i*; *μ* = overall mean; *T_i_* = fixed effect of diet treatment; *ε_ij_* = random error obeying a normal distribution N(0, *σ*^2^). Tukey pairwise comparisons were performed when the treatment effect was significant (*p* < 0.05).

Linear and quadratic polynomial regression analyses were performed to characterize dose–response relationships between FML inclusion levels and all measured indicators.

Linear regression: *Y* = *a* + *bX* + *ε*

Quadratic regression: *Y* = *a* + *bX* + *cX*^2^ + *ε*

*Y* = detected index value; *X* = the dietary FML inclusion level; *a* = intercept; *b* = linear coefficient; *c* = quadratic coefficient; *ε* = random error. The coefficient of determination (*R*^2^) evaluated model fitness. A significant quadratic coefficient (*c*, *p* < 0.05) indicated nonlinear trends. An extra sum-of-squares F-test compared the fitting performance of linear and quadratic models; *p* < 0.05 indicated that the quadratic model fitted better. The universal significance threshold was *p* < 0.05.

## 3. Results

### 3.1. Growth Performance and Morphometric Parameters

FBW, WGR, SGR, FCR, FR, SR, CF, VSI, and HSI are shown in Table 1. One-way ANOVA analysis showed that after 10 weeks of feeding, compared with the control group, FML6 significantly reduced the FBW, WGR, SGR, and HSI of sturgeon and significantly increased FCR (*p* < 0.05); however, no significant differences were observed in FR, SR, CF, or VSI among all groups (*p* > 0.05).

### 3.2. Changes in Serum Antioxidant-Related Indices and Immune Parameters

**Figure 1:**
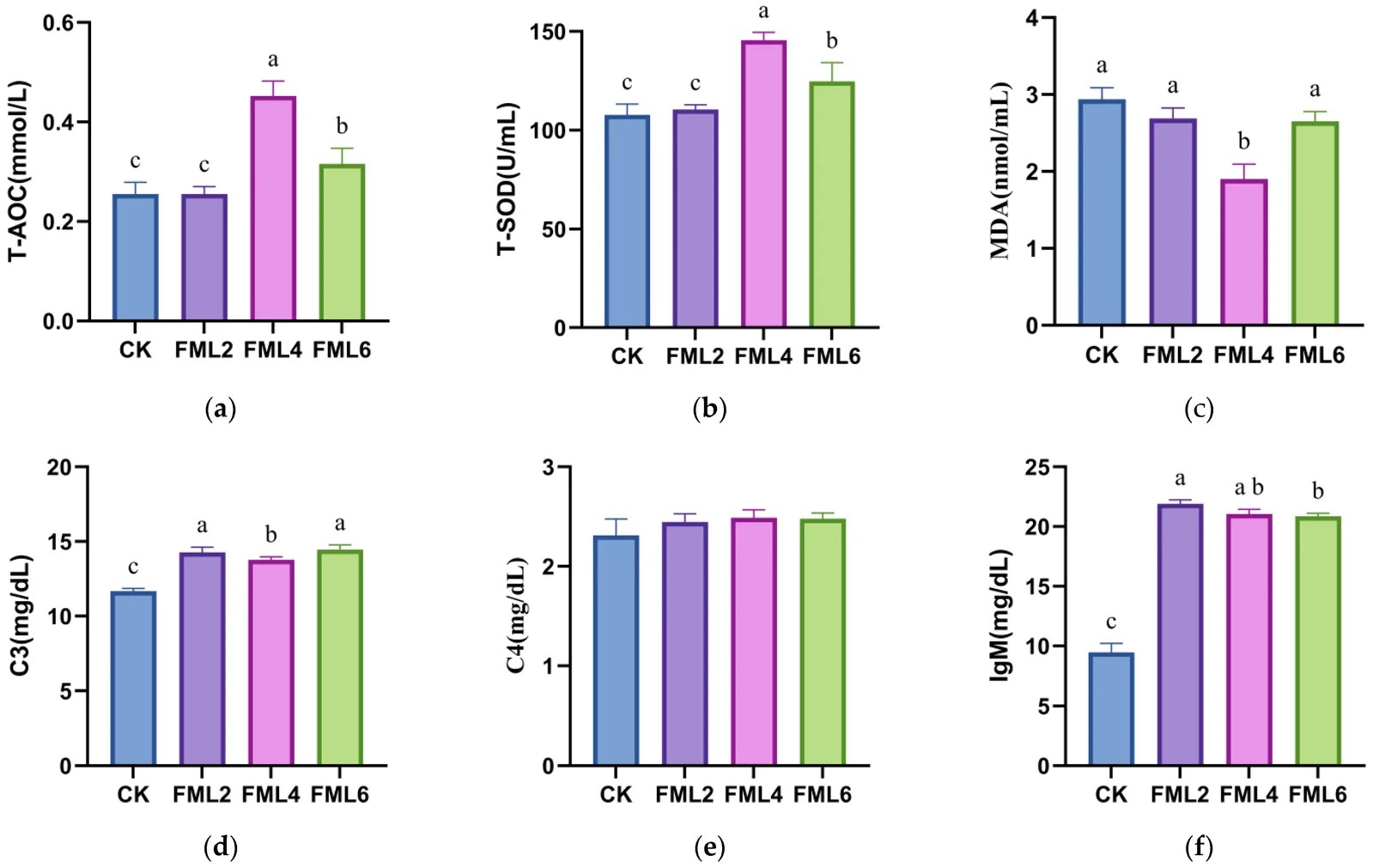
Effects of FML on serum antioxidant indices and immune parameters of *Acipenser schrenckii*. (**a**–**c**) Serum antioxidant indicators: (**a**) T-AOC level; (**b**) T-SOD activity; (**c**) MDA content. (**d**–**f**) Serum immune indices: (**d**) C3 concentration; (**e**) C4 concentration; (**f**) IgM concentration. All data are expressed as mean ± SEM. Columns marked with distinct lowercase letters are significantly different among groups (*p* < 0.05).

Serum antioxidant-related indices and immune parameters are summarized in Figure 1. Relative to the CK group, FML treatment increased serum T-AOC and T-SOD activities and decreased MDA concentration. Significant elevations in T-AOC and T-SOD were observed in the FML4 and FML6 groups (*p* < 0.05), whereas MDA was significantly reduced in the FML4 group (*p* < 0.05). All FML treatments induced significant increases in serum C3 and IgM concentrations (*p* < 0.05), while serum C4 showed no significant differences among all treatments (*p* > 0.05).

Linear and quadratic polynomial regression analyses were performed to evaluate the dose-dependent responses of serum indices to graded FML treatment. Significant linear relationships were detected for T-AOC, T-SOD, and C4 (*p* < 0.05), whereas the quadratic terms were non-significant. By contrast, significant quadratic responses were observed for serum MDA, C3, and IgM (*p* < 0.05). Based on the established quadratic models, the FML inclusion level to obtain the minimum serum MDA concentration was estimated to be 3.66%. The predicted inclusion levels corresponding to the maximum concentrations of serum C3 and IgM were 4.62% and 4.06%, respectively.

### 3.3. Changes in Intestinal Digestive Function and Morphology

**Figure 2:**
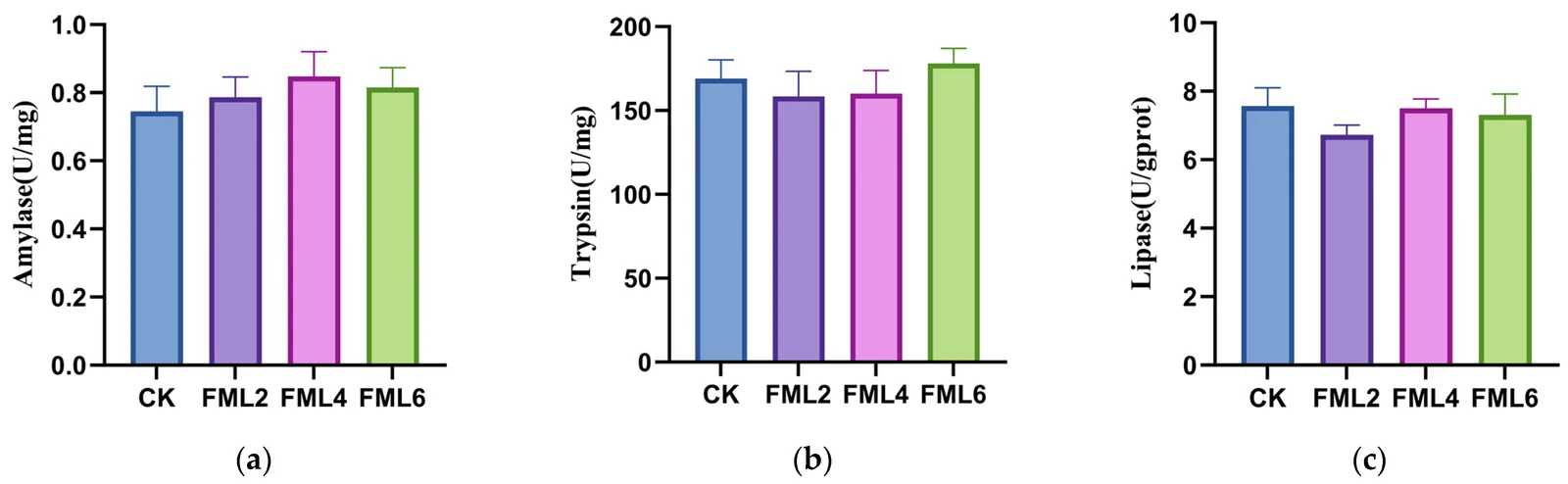

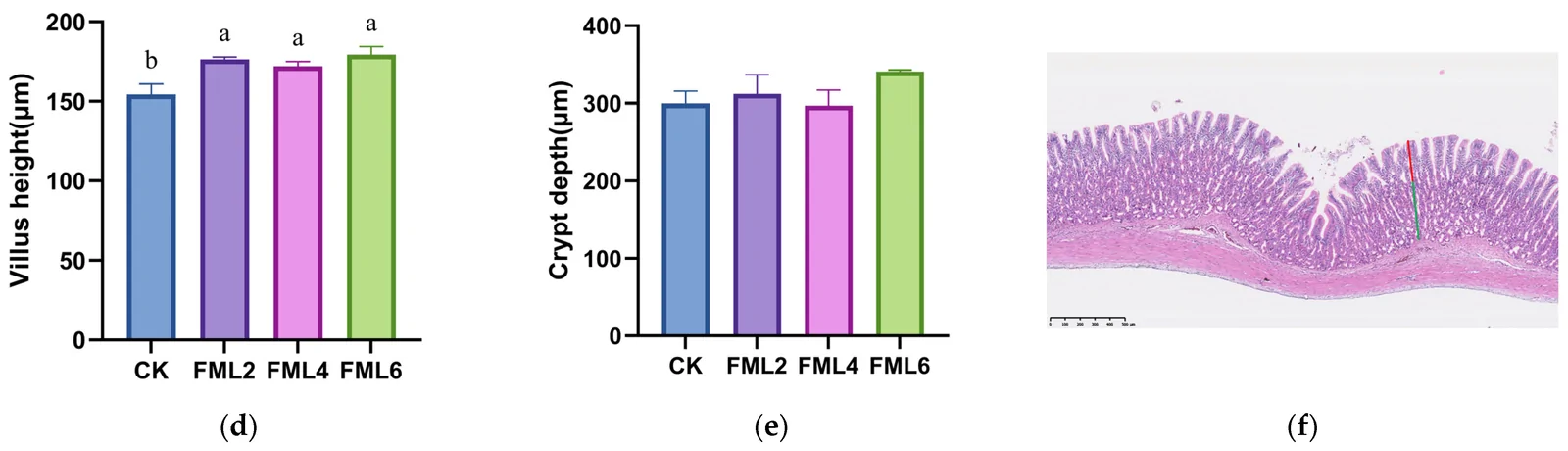
Effects of dietary FML inclusion on intestinal digestive enzymes and morphology of *Acipenser schrenckii*. (**a**–**c**) Intestinal digestive enzymes: (**a**) amylase activity; (**b**) trypsin activity; (**c**) lipase activity; (**d**–**f**) Intestinal morphological indexes: (**d**) villus height; (**e**) crypt depth; (**f**) representative HE histological micrographs of intestinal tissue. Red lines indicate villus height, and green lines indicate crypt depth. Data are presented as mean ± SEM. Values with different lowercase letters indicate significant differences between groups (*p* < 0.05).

Compared with the CK group, no significant differences were observed in digestive enzymes, including amylase, lipase, and trypsin (Figure 2a–c, *p* > 0.05). Villus height was significantly higher in FML treatments and exhibited a significant linear increasing trend with rising FML inclusion levels (Figure 2d, *p* < 0.05). Crypt depth showed no significant differences among all groups (Figure 2e, *p* > 0.05). Although villus height increased linearly with FML inclusion, the calculated villus height-to-crypt depth ratio did not reach statistical significance between the FML6 and CK groups (Tukey’s test, *p* = 0.0568), indicating that the enhanced villus height was largely offset by the numerically higher crypt depth at the 6% inclusion level.

### 3.4. Intestinal Microbial Community Composition and Diversity of Acipenser schrenckii

#### 3.4.1. Alpha and Beta Diversity Analysis

**Figure 3:**
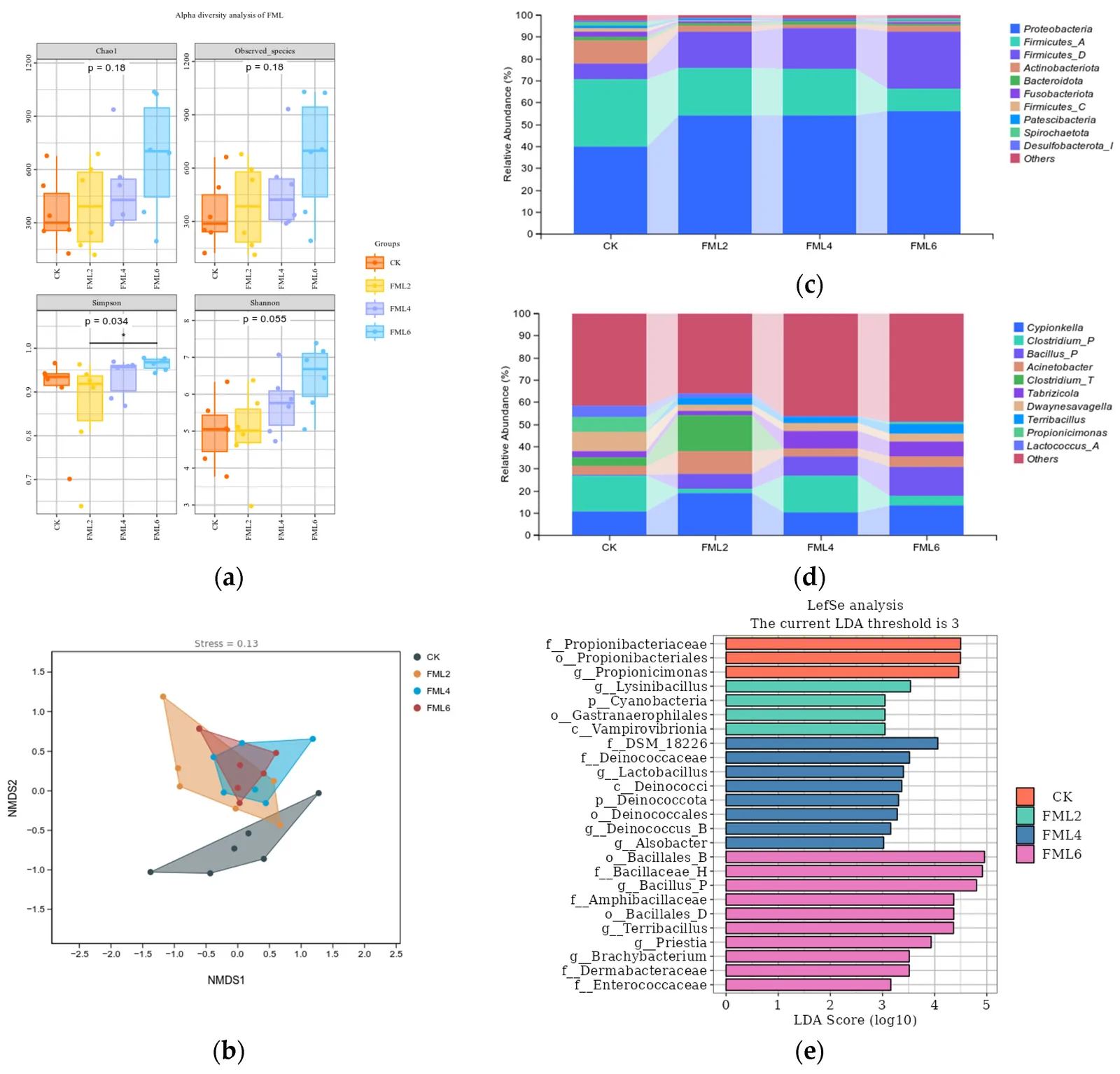
Effects of FML on intestinal microbial diversity and community composition. (**a**) Alpha diversity indices of intestinal microbiota among different groups. (**b**) Beta diversity revealed by NMDS analysis. (**c**) Microbial community composition at the phylum level. (**d**) Microbial community composition at the genus level. (**e**) Group-specific differential microbial biomarkers identified by LEfSe analysis. * *p* < 0.05.

No significant differences in any alpha diversity indices were detected in FML groups compared with the CK group (*p* > 0.05). Within the FML groups, a significant difference in the Simpson index was observed between FML2 and FML6 (Figure 3a, *p* < 0.05). Stress values of both NMDS ordinations were less than 0.2, confirming the reliability of the ordination results (Figure 3b). Adonis analysis identified a significant global divergence in the overall structure of intestinal microbial communities across FML treatments (*p* = 0.013).

#### 3.4.2. Species Composition Analysis

Community composition analyses of the top 10 intestinal microbial taxa were performed at the phylum and genus levels. The results revealed that Proteobacteria and Firmicutes (including Firmicutes_A, Firmicutes_D, and Firmicutes_C) were the dominant core phyla shared by all groups. The relative abundances of Proteobacteria and Firmicutes_D increased in a dose-dependent manner with increasing dietary FML inclusion levels, whereas the relative abundance of Actinobacteriota decreased (Figure 3c).

At the genus level, *Clostridium_T* and *Acinetobacter* were significantly enriched in the FML2 group. The relative abundances of *Bacillus_P* and *Tabrizicola* exhibited an increasing trend along with elevated FML treatment, reaching the highest values in the FML6 group (Figure 3d).

#### 3.4.3. Identification of Potential Intestinal Microbial Biomarkers

*Lysinibacillus* (phylum Firmicutes_D) and several taxa belonging to Cyanobacteria were significantly enriched in the FML2 group. The number of differentially enriched taxa gradually increased as dietary fermented mulberry leaf inclusion increased. The FML4 group was distinguished by the enrichment of f_DSM_18226 and *Lactobacillus* within Firmicutes_D, along with multiple taxa assigned to the phylum Deinococcota. The microbial biomarkers distinguishing the FML6 group mainly belonged to Firmicutes_D, consisting of a series of Bacillales-associated potential probiotic taxa, including *Bacillus_P*, *Terribacillus*, and *Priestia* (Figure 3e).

### 3.5. Untargeted Metabolomics Analysis of Differential Metabolites

#### 3.5.1. Screening for Differential Metabolites

**Figure 4:**
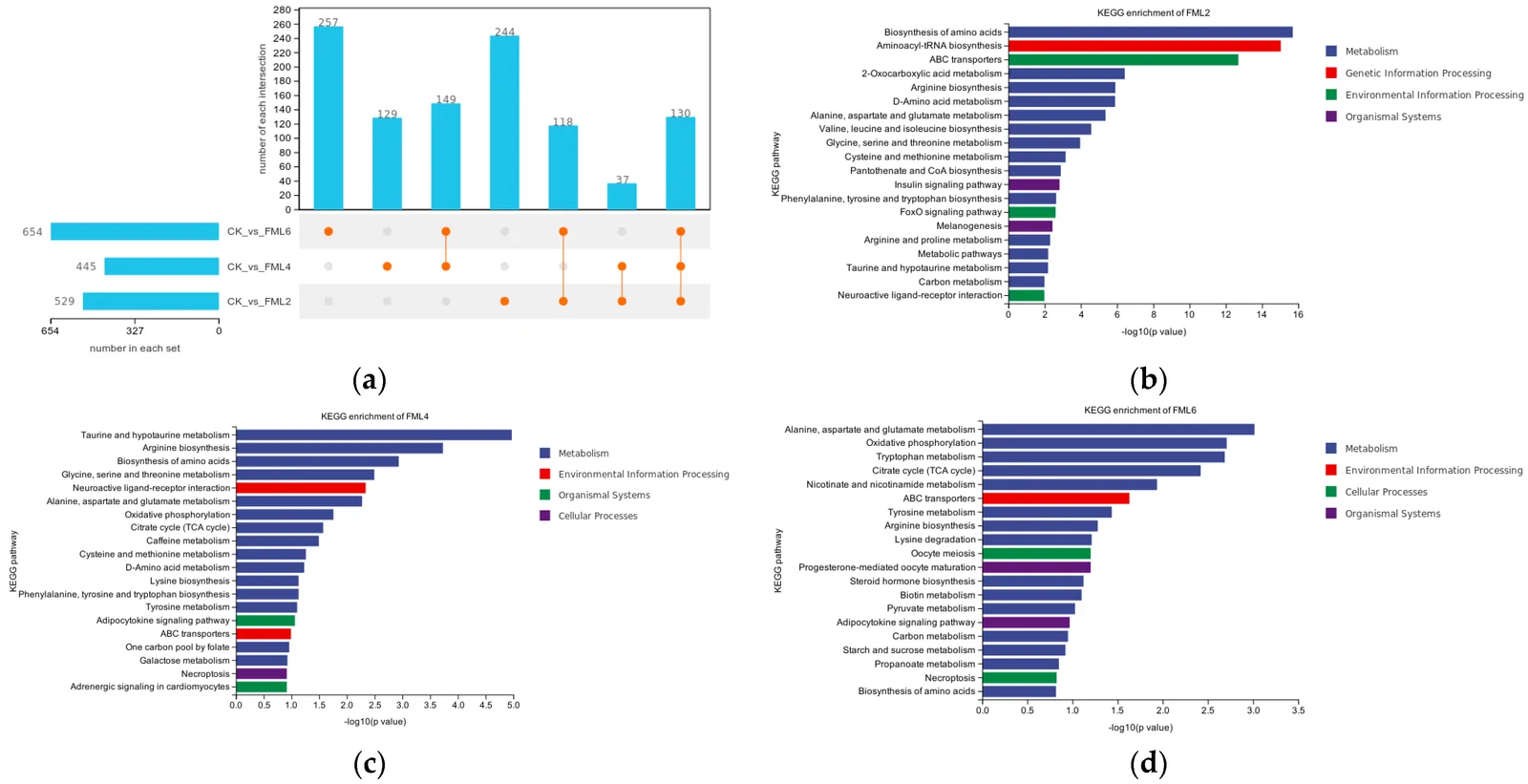
Differential metabolite distribution and KEGG pathway enrichment analysis. (**a**) UpSet plot showing the distribution of shared and unique differential metabolites among comparison groups. (**b**–**d**) KEGG pathway enrichment analysis of differential metabolites derived from the comparisons of CK vs. FML2 (**b**), CK vs. FML4 (**c**), and CK vs. FML6 (**d**), respectively. The *x*-axis represents −log 10 (*p*-value), and the *y*-axis represents the KEGG pathway.

Compared with the CK group, 529, 445, and 654 differential metabolites were identified in the FML2, FML4, and FML6 groups, respectively, among which 244, 129, and 257 metabolites were uniquely expressed in each corresponding comparison group. A total of 130 metabolites were commonly identified as differential in all three FML dosage groups (intersection of the three groups, Figure 4a), based on the criteria of VIP > 1.0 and *p* < 0.05. Thirty metabolites were consistently downregulated, including one alkaloid and derivative, three benzenoids, five lipids and lipid-like molecules, three organic acids and derivatives, three organic oxygen compounds, five organoheterocyclic compounds, and four phenylpropanoids and polyketides. Ninety-eight metabolites were continuously upregulated, covering 3 alkaloids and derivatives, 6 benzenoids, 16 lipids and lipid-like molecules, 27 organic acids and derivatives, 9 organic oxygen compounds, 14 organoheterocyclic compounds, and 4 phenylpropanoids and polyketides. Representative differential metabolites across the three FML dosage groups included methoxytyrosine (VIP > 1.9, FC > 1.6) and diethyl glutamate (VIP > 1.6, FC > 1.6), which were consistently upregulated in all FML treatments. In the FML4 and FML6 groups, two Maillard reaction products, 4-(carboxymethyl)phenylalanine (FML4: VIP = 2.05, FC = 1.86; FML6: VIP = 1.70, FC = 2.05) and N-(1-deoxy-1-fructosyl)phenylalanine (FML4: VIP = 1.85, FC = 2.23; FML6: VIP = 1.75, FC = 3.70), showed marked dose-dependent increases. In the FML6 group, TCA cycle intermediates, including citric acid (VIP = 1.42, FC = 2.06) and succinic acid (VIP = 1.08, FC = 1.39) were significantly upregulated, while L-5-hydroxytryptophan (VIP = 1.23, FC = 0.62) was downregulated, suggesting perturbations in energy metabolism and tryptophan catabolism at the highest inclusion level. These metabolic shifts may partly explain the growth depression observed at 6% FML inclusion.

#### 3.5.2. KEGG Pathway Enrichment Analysis

In the FML treatment set, the enriched pathways were predominantly concentrated on amino acid metabolism and core energy metabolism. For FML2, the top enriched pathways included biosynthesis of amino acids, aminoacyl-tRNA biosynthesis, and ABC transporters (Figure 4b). In FML4, taurine and hypotaurine metabolism, arginine biosynthesis, and energy-related pathways such as the TCA cycle and oxidative phosphorylation were most prominent (Figure 4c). In FML6, alanine, aspartate, and glutamate metabolism, oxidative phosphorylation, and tryptophan metabolism were significantly enriched (Figure 4d).

### 3.6. Metabolite Modules and Microbe–Metabolite Correlations

**Figure 5:**
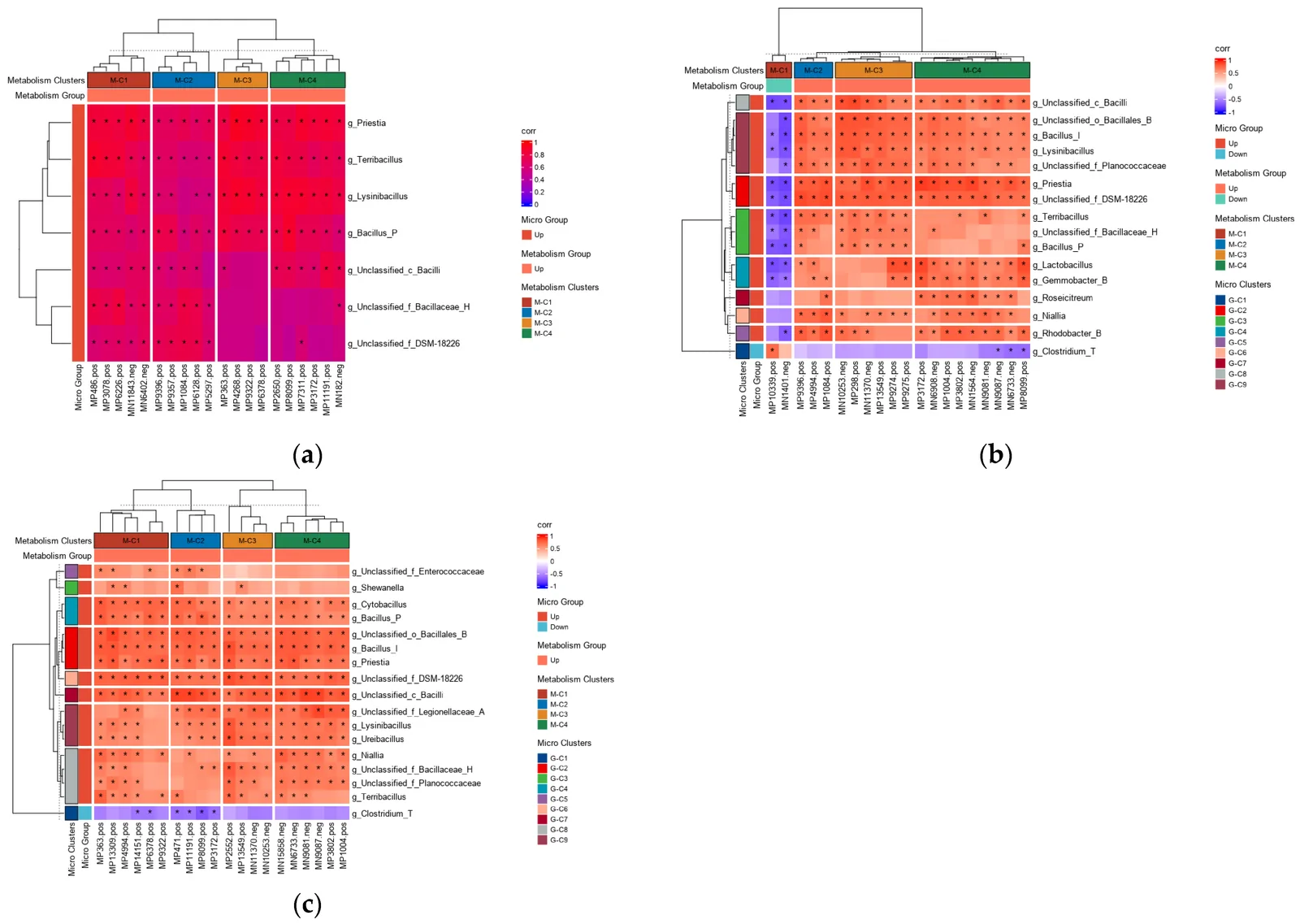
Correlation heatmap of differential gut microbial genera and metabolites under graded FML inclusion. (**a**) FML2 vs. CK; (**b**) FML4 vs. CK; (**c**) FML6 vs. CK. The horizontal axis represents differential gut microbial genera at the genus level, and the vertical axis represents differential metabolites. Red color indicates significant positive correlations, while blue–purple color indicates significant negative correlations. Asterisks denote statistically significant correlations (*p* < 0.05).

Spearman correlation analysis was performed between the relative abundance of differential bacterial genera and the intensity of differential metabolite modules, generating a correlation matrix for each FML dosage group. Core *Bacillus* taxa, including *Priestia*, *Terribacillus*, *Lysinibacillus*, and *Bacillus_P*, displayed consistently positive correlations with the majority of upregulated metabolite modules (M-C1 to M-C4) across FML2, FML4, and FML6 (Figure 5a–c). For example, correlations between M-C1 and *Priestia* ranged from r = 0.62 to 0.92 (*p* < 0.05) across all three dosage groups. By contrast, *Clostridium_T* was the primary taxon negatively associated with activated metabolic pathways in FML4 and FML6, with correlation coefficients ranging from r = −0.57 to −0.73 (*p* < 0.05). Given the relatively small sample size (*n* = 6 per group), these correlation analyses have limited statistical power and should be interpreted as exploratory; the most robust associations are those consistently observed across at least two dosage groups.

### 3.7. RNA Isolation, Reverse Transcription, and mRNA Analysis

#### 3.7.1. Antioxidant-Related Gene Expression

**Figure 6:**
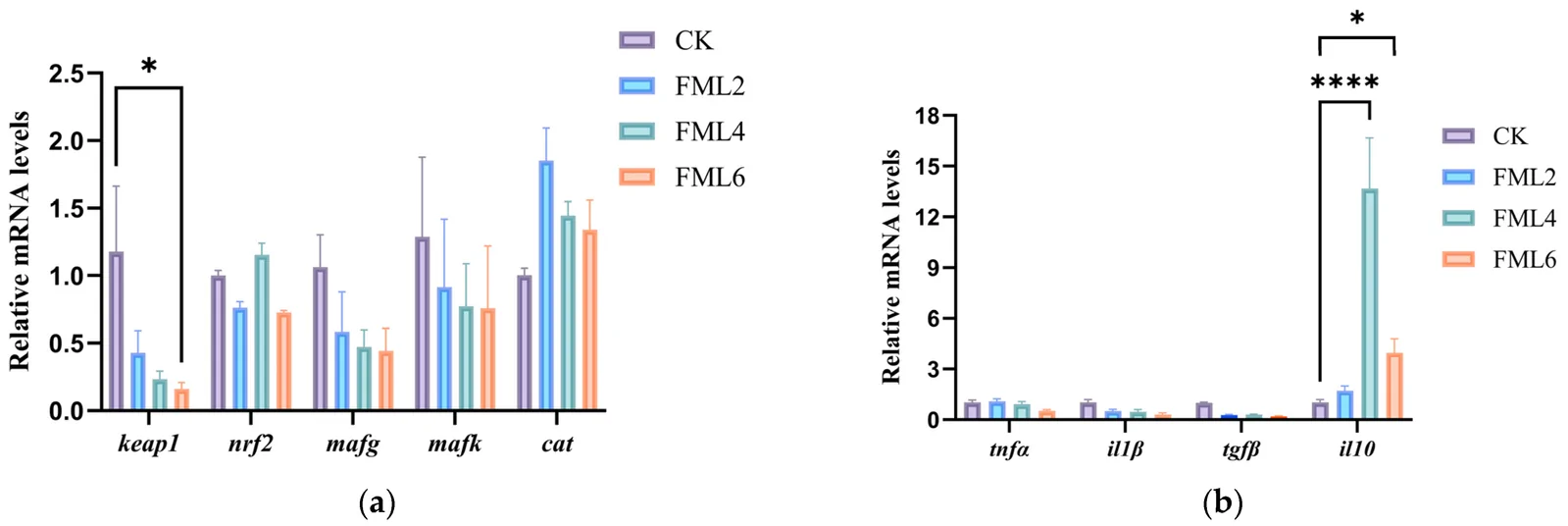
Antioxidant and immune-related gene expression of *Acipenser schrenckii*. (**a**) Antioxidant-related genes (*keap1*, *nrf2*, *mafg*, *mafk*, *cat*); (**b**) Immune and inflammatory cytokine genes (*tnfα*, *il1β*, *tgfβ*, *il10*). Data are presented as mean ± SEM, error bars indicate SEM.* and **** represent significant differences at *p* < 0.05 and *p* < 0.0001, respectively.

The transcriptional profiles of core genes involved in the Nrf2/Keap1 antioxidant pathway were determined (Figure 6a). Compared with the CK group, *keap1* mRNA expression was downregulated in all FML treatments, with a significant reduction observed in the FML6 group (*p* < 0.05). The transcription factor gene *nrf2* and its cofactor genes *mafg* and *mafk* were slightly downregulated in experimental groups, while *cat* showed a slight upregulation; however, none of these changes reached statistical significance.

#### 3.7.2. Immune-Related Gene Expression

Intestinal immune and inflammatory gene expression was further analyzed (Figure 6b). The pro-inflammatory genes *tnfα* and *il1β*, as well as the immunomodulatory gene *tgfβ*, exhibited no significant differences across all treatments. By comparison, the anti-inflammatory cytokine gene *il10* was significantly upregulated in the FML4 and FML6 groups (*p* < 0.05), and the maximum expression level of *il10* was detected in the FML4 group (*p* < 0.0001).

## 4. Discussion

### 4.1. Effects of FML on the Growth Performance and Digestive Function of Amur Sturgeon

The present study innovatively applied fermentation technology to improve the utilization efficiency of mulberry leaves. Previous research has indicated that high inclusion levels of raw mulberry leaves exert adverse impacts on animal growth. For example, dietary supplementation with 15% mulberry leaves significantly impaired the growth performance of finishing pigs [28]. In contrast, appropriate inclusion of fermented mulberry leaves has been confirmed to enhance the growth performance of aquatic animals, such as golden pompano and Chinese giant salamander [29,30]. Consistent with these reports, the graded replacement trial in this study revealed that low and moderate FML inclusion (2% and 4%) sustained growth comparable to the control group under isonitrogenous and isoenergetic conditions, whereas the highest inclusion level (6%) induced obvious growth depression, indicating a dose-dependent negative effect of excessive plant-derived ingredients when replacing fishmeal. Notably, the optimal replacement level of 4% obtained in this study was markedly lower than the suitable raw mulberry leaf inclusion (30.95–65%) reported for omnivorous carps [18,31,32], but was consistent with the optimal 4–6% mulberry leaf levels for other carnivorous and omnivorous fish, such as golden pompano and crucian carp [29,33]. Such interspecies differences in suitable dosages are likely associated with variations in feeding habits and physiology. Sturgeon and golden pompano are typical carnivores, while carps are omnivorous fish, which leads to inherent differences in intestinal microbiota configuration, nutrient metabolism capacity, and tolerance to plant feedstuffs [34].

Intestinal morphology is a key factor governing nutrient digestion and absorption in aquatic animals, and villus height (Vh) and crypt depth (Cd) are widely adopted core indicators. Increased villus height enlarges the contact surface between the intestinal epithelium and digesta, facilitating sufficient digestion and efficient nutrient absorption [35]. In this study, intestinal villus height was significantly elevated in all FML replacement groups, demonstrating that fermented mulberry leaves exert beneficial regulatory effects on intestinal mucosal morphology even when replacing a portion of fishmeal. Meanwhile, FML inclusion did not alter the activities of intestinal digestive enzymes, suggesting that the improvements in growth and morphology were not attributable to enhanced enzymatic digestion. Nevertheless, these beneficial morphological improvements could not offset the growth inhibition observed at the excessive 6% inclusion level. Taken together, these findings confirm that fermentation technology effectively converts raw mulberry leaves into a viable feed ingredient for carnivorous fish, with moderate inclusion (2–4%) offering a balanced improvement in intestinal health and growth performance.

### 4.2. FML Can Enhance the Antioxidant Capacity of Acipenser schrenckii

Mulberry leaves contain abundant bioactive polysaccharides and flavonoids with potent free radical-scavenging and antioxidative properties [36,37]. In aquatic species, serum T-AOC, T-SOD, and MDA serve as widely adopted biomarkers for evaluating whole-body antioxidant defense and oxidative stress levels [38]. In the present study, graded FML inclusion induced significant quadratic dose-dependent antioxidant responses in sturgeon, with maximum effects achieved at moderate inclusion levels. The quadratic model predicted that the FML level corresponding to the minimum MDA concentration was 3.66%. These results indicate that appropriate FML inclusion effectively enhances antioxidant defenses, whereas excessive inclusion gradually diminishes these benefits and permits rebound oxidative stress.

At the molecular level, the K*eap1*–N*rf2* signaling pathway acts as the core regulatory axis governing cellular antioxidant responses [39]. Significant downregulation of keap1 expression across all FML groups suggests that FML may alleviate Keap1-mediated inhibition of Nrf2, potentially facilitating the downstream antioxidant cascade. Consistent with this observation, CAT activity showed an overall increasing trend in FML treatments, supporting the activation of the enzymatic antioxidant system. These molecular changes were paralleled by improvements in serum antioxidant parameters, as reflected by elevated T-AOC and T-SOD activities and reduced MDA concentrations in moderate FML groups [40].

In summary, FML enhances the antioxidant capacity of Amur sturgeon by activating the K*eap1*–N*rf2* antioxidant pathway and stimulating key antioxidant enzymes. Nevertheless, the observed quadratic dose effects indicate that high levels of FML exceed the optimal regulatory threshold, resulting in attenuated antioxidant benefits and cumulative oxidative damage.

### 4.3. FML Enhances Immune Function of Acipenser schrenckii

Immunoglobulins constitute core effector molecules of humoral immunity in fish. Acipenseridae express an IgM homolog (~90 kDa) that governs systemic humoral defense and fulfills central functions in pathogen recognition and elimination [41]. C3 and C4 are also essential upstream components of the complement cascade, jointly mediating inflammatory reactions and pathogen lysis [42]. In the present study, serum IgM and C3 concentrations were significantly increased in FML treatments. Furthermore, these serum immune parameters displayed distinct quadratic dose-dependent responses with clear optimal inflection points. The predicted FML inclusion levels maximizing C3 and IgM concentrations were 4.62% and 4.06%, respectively. These consistent quadratic trends suggest that immune enhancement is only achieved within a moderate dosage window, whereas high-dose inclusion gradually impairs humoral immune capacity.

Intestinal immune homeostasis relies ona dynamic equilibrium between pro-inflammatory and anti-inflammatory cytokine networks [43]. The pro-inflammatory mediators IL-1β and TNF-α initiate the TAK1/MAPK/NF-κB signaling cascades and exacerbate excessive intestinal inflammatory damage [44,45]. In contrast, IL-10 serves as a pivotal anti-inflammatory cytokine, which suppresses hyperactivated inflammatory signaling and maintains intestinal mucosal integrity [46]. In this experiment, no significant intergroup differences were observed in the transcript levels of pro-inflammatory *tnfα*, *il1β,* and immunomodulatory *tgfβ*, indicating that FML inclusion did not induce aberrant intestinal inflammatory stress. Meanwhile, the anti-inflammatory gene *il10* was significantly upregulated in the FML4 and FML6 groups, with peak transcription detected in FML4. Such selective induction of *il10*, without concurrent upregulation of pro-inflammatory genes, demonstrates that moderate FML inclusion remodels intestinal immune balance rather than merely suppressing or broadly stimulating inflammatory responses [47].

In summary, moderate FML inclusion enhanced systemic humoral immunity by elevating serum IgM and complement levels and stabilized intestinal immune homeostasis through specific induction of the anti-inflammatory cytokine gene *il10*. The optimal inclusion levels for immune traits calculated from quadratic regression were concentrated within the range of 4.06–4.62%. These findings further support that moderate FML replacement represents the optimal dosage for balancing growth, antioxidant capacity, and immune function in carnivorous Amur sturgeon.

### 4.4. FML Regulates the Intestinal Microbiota

Gut microbiota in aquatic animals are jointly modulated by host-derived intrinsic factors and external environmental stimuli. Dietary components constitute one of the most pivotal drivers that remodel the compositional and functional profiles of intestinal microbiota [48]. In accordance with previous investigations on sturgeon, Proteobacteria, Firmicutes, Bacteroidetes, and Actinobacteria were identified as the dominant core phyla of *Acipenser schrenckii* intestinal microbiota [49,50]. Alpha diversity remained comparable across all treatments, indicating that partial replacement of fishmeal with fermented mulberry leaf (FML) did not disrupt the fundamental richness and evenness of intestinal microbial communities. However, beta diversity analysis revealed clear dose-dependent microbial separation, demonstrating that graded FML inclusion substantially altered the overall intestinal bacterial community structure.

Dietary FML comprehensively remodeled the gut microbiota and selectively enriched multiple beneficial probiotic genera, including *Bacillus*, *Lysinibacillus*, *Clostridium_T*, and *Tabrizicola*. These Firmicutes-derived beneficial taxa are well recognized for strengthening intestinal barrier integrity, promoting mucosal repair, and inhibiting the proliferation of opportunistic pathogens [51,52]. In the present study, FML induced distinct dose-dependent microbial succession patterns. The low-dose FML2 group was specifically characterized by significant enrichment of *Clostridium_T* and *Lysinibacillus*, which primarily contribute to maintaining intestinal immune homeostasis and stabilizing mucosal morphology [42,53]. In contrast, increased FML inclusion continuously enriched *Bacillus_P*, *Terribacillus*, and *Priestia*, with the highest abundance observed in the FML6 group. These gradient microbial shifts suggested that low-dose FML tended to optimize intestinal immune and structural stability, while high-dose FML specifically enriched Bacillales-related probiotics with strong metabolic and antioxidant regulatory potential [54,55].

The progressive enrichment of beneficial microbial taxa across FML treatments provides a consistent microbial explanation for the improved intestinal villus morphology, enhanced antioxidant capacity, and balanced intestinal inflammatory status observed in FML-supplied groups [56]. Collectively, these findings demonstrate that FML inclusion remodeled gut microbiota in a dose-dependent manner, selectively enriching versatile probiotic communities and restructuring intestinal microbial ecology. Such microbiota optimization synergistically contributed to improved intestinal barrier function, antioxidant defense, and immune homeostasis, thereby supporting the overall intestinal health and metabolic performance of *Acipenser schrenckii*.

### 4.5. FML Regulates Intestinal Metabolism

Metabolomics enables the characterization of diet-provoked metabolic shifts in aquatic animals [57,58]. Intestinal metabolic signatures reflect overall nutrient utilization efficiency and reciprocal host–microbe metabolic dialog, which modulates gut physiological conditions [59,60]. In the present study, KEGG pathway enrichment analysis demonstrated that differentially expressed metabolites induced by FML inclusion were primarily concentrated in amino acid metabolism and ABC transporter pathways, two core functional modules governing intestinal physiological homeostasis in fish.

Amino acid metabolism is essential for sustaining cellular metabolism, protein turnover, and immune function in aquatic species [61]. In particular, arginine metabolism serves as a central metabolic hub coordinating protein synthesis, carbohydrate catabolism, and the generation of multiple bioactive signaling molecules [62]. Activated arginine biosynthesis and downstream metabolic cascades alleviate glycolipid metabolic disturbances, enhance systemic antioxidant defense, and strengthen host immune competence [63,64]. In parallel, ABC transporters constitute an energy-dependent transmembrane transport system that mediates nutrient uptake, endogenous substance trafficking, and immune modulation, thereby maintaining cellular homeostasis and environmental adaptability [65]. Accordingly, the simultaneous activation of amino acid metabolism and ABC transporter pathways provides a critical metabolic foundation for the improved antioxidant capacity and humoral immune performance observed in FML-treated sturgeon.

Notably, FML induced distinct dose-dependent metabolic divergence in the intestinal metabolome. Low-dose FML2 predominantly promoted amino acid biosynthesis pathways, which contributed to balanced nutrient utilization and basic intestinal physiological stability. In comparison, medium (FML4) and high-dose (FML6) inclusion further significantly upregulated central energy metabolism pathways, including the TCA cycle and oxidative phosphorylation. Enhanced energy metabolism flux further fortified intestinal antioxidant and immune homeostasis [66], which closely corresponded to the elevated serum T-AOC, SOD, C3, and IgM levels in moderate and high FML groups. Collectively, these dose-dependent metabolic shifts illustrate that FML inclusion remodels intestinal metabolic patterns from amino acid-oriented utilization at low doses to energy-metabolism reinforcement at moderate-to-high doses, thereby supporting enhanced intestinal health and immune function.

### 4.6. FML Reshapes Distinct Microbe-Metabolite Interactive Networks in the Intestine

Dietary nutrients entering the intestinal tract are fermented and degraded by indigenous gut microbiota, yielding a variety of bioactive metabolites that mediate host intestinal physiological functions [67]. In line with this core microbe–nutrient metabolic axis, the present study identified distinct correlation patterns between intestinal microbial taxa and metabolic profiles under FML inclusion.

In FML-treated groups, core beneficial genera belonging to Bacillales, including *Priestia*, *Terribacillus*, and *Bacillus_P,* exhibited extensive positive correlations with key intestinal metabolic modules. These spore-forming probiotic taxa are critically involved in promoting intestinal amino acid biosynthesis and enhancing mitochondrial energy metabolism [68], which is consistent with the enriched amino acid metabolism, TCA cycle, and oxidative phosphorylation pathways observed in the FML metabolome. Furthermore, these Bacillales-related taxa serve as vital mediators of host antioxidant capacity and immune homeostasis through bidirectional microbe–host metabolic crosstalk [54]. This mechanistic interaction likely contributes to the improved serum antioxidant status and enhanced humoral immune parameters in FML-treated sturgeon.

Collectively, dietary FML inclusion reshaped the intestinal microbial community by enriching Bacillales-dominated probiotic populations, which in turn drove the reprogramming of amino acid and energy metabolism. This coordinated microbial–metabolic remodeling ultimately supported improved intestinal barrier function, antioxidant defense, and immune performance in Amur sturgeon.

## 5. Limitations

Several limitations should be acknowledged. First, juvenile sturgeon of mixed sex were used, as sex cannot be determined externally at this stage, which may contribute to inter-individual variability. Second, for serum biochemical indices, intestinal digestive enzyme activities, and qPCR analyses, samples were derived from three fish representing three of the four replicate cages (one fish per cage); inter-cage variation for these parameters may therefore be underestimated. Third, KEGG pathway annotation relied on the closely related species *Acipenser ruthenus* due to the lack of a well-annotated *A. schrenckii* genome, making pathway assignments approximate. Fourth, the 10-week trial duration and the maximum inclusion level of 6% limit our understanding of long-term effects and higher-dose responses. Fifth, the microbiota–metabolite correlations are associative rather than causal, and functional validation is needed. Finally, the modest sample size (*n* = 6 per group) for omics analyses limits statistical power; accordingly, these results should be considered exploratory.

## 6. Conclusions

This study systematically explored dose-dependent impacts of dietary fermented mulberry leaf (FML) on growth performance, intestinal health, antioxidant-immune status, as well as gut microbial and metabolic characteristics in *Acipenser schrenckii*. Moderate FML supplementation preserved intestinal morphological integrity, elevated systemic antioxidant capacity, and mitigated intestinal oxidative stress, with Keap1 downregulation suggesting potential activation of the Keap1–Nrf2 cascade. It also improved humoral immune responses and maintained intestinal immune homeostasis without triggering inflammatory reactions.

Multi-omics analyses confirmed that FML triggered pronounced dose-dependent remodeling of the intestinal microecosystem. Moderate FML favored the proliferation of beneficial gut bacteria and modulated intestinal metabolic functional profiles, which were closely associated with improved host antioxidant and immune phenotypes. Quadratic regression suggested the optimal functional dosage of FML ranged from 3.66% to 4.62%. Although high-dose FML still exerted favorable modulatory effects on gut microecology, excessive inclusion suppressed fish growth. Quadratic regression based on immune and antioxidant parameters suggested an optimal FML dosage range of 3.66–4.62%. However, since growth performance did not show a statistically definable optimum, the 4% recommendation rests on non-inferior growth combined with peak immune and antioxidant responses, rather than on growth superiority.

Collectively, FML represents a promising sustainable feed ingredient for sturgeon culture. This paper provides new theoretical evidence supporting the value of mulberry leaf agricultural by-products for sustainable nutrition in carnivorous fish.

## Figures and Tables

**Table 1:** Effects of FMLs on the growth performance and morphometric parameters in Amur sturgeon (means ± SEM).

Items	Groups				SEM	*p*-Value		
	CK	FML2	FML4	FML6		ANOVA	Linear	Quadratic
Growth performance								
FBW (g)	421.05 ^a^	405.43 ^ab^	405.20 ^ab^	384.98 ^b^	6.6933	0.0050	0.0013	0.7229
WGR (%)	112.48 ^a^	101.41 ^ab^	104.00 ^ab^	94.15 ^b^	3.0512	0.0013	0.0015	0.8463
SGR (%/d)	1.08 ^a^	1.00 ^ab^	1.02 ^ab^	0.95 ^b^	0.0214	0.001	0.0015	0.9102
FCR	1.26 ^a^	1.39 ^ab^	1.36 ^ab^	1.49 ^b^	0.0434	0.0101	0.0014	0.9754
FR (%)	1.28	1.33	1.32	1.35	0.0212	0.4237	0.0207	0.4255
SR (%)	95.00	95.00	95.00	95.00	NA	NA	NA	NA
Morphometric parameters								
CF (g/cm^3^)	0.76	0.71	0.71	0.73	0.0238	0.0657	0.0681	0.2608
HSI (%)	3.74 ^a^	3.38 ^ab^	3.49 ^ab^	3.03 ^b^	0.2163	0.0271	0.0077	0.7531
VSI (%)	10.22	9.75	10.34	9.49	0.4266	0.4589	0.2617	0.1757

FBW = final body weight; WGR = weight gain rate; SGR = specific growth rate; FCR = feed conversion rate; FR = Feeding rate; SR = Survival rate; CF = Condition factor; HSI = Hepatosomatic index; VSI = Viscerosomatic index. ^a,b^ Within a row, means without a common superscript letter differ at *p* < 0.05, *n* = 4.

## Data Availability

The original contributions presented in this study are included in the article/Supplementary Material. Further inquiries can be directed to the corresponding authors.

## Supplementary Materials

The following supporting information can be downloaded at: https://www.mdpi.com/article/10.3390/ani16182918/s1, Table S1: Proximate nutrients and tannin concentrations of ML and FML (% dry matter). Table S2: Formulation and compositions of experimental diets (%). Table S3: Amino acid composition of experimental diets (g/100 g crude protein). Table S4: Primer sequences for real-time PCR.

## Abbreviations

The following abbreviations are used in this manuscript:

FML	Fermented mulberry leaves
T-AOC	Total antioxidative capability
T-SOD	Total superoxide dismutase
MDA	Malondialdehyde
C3	Complement 3
C4	Complement 4
IgM	Immunoglobulin M
Vh	Villus height
Cd	Crypt depth
*keap1*	Kelch-like ECH-associated protein 1
*nrf2*	Nuclear Factor Erythroid 2-Related Factor 2
*mafk*	MAF bZIP Transcription Factor K
*maf**g*	MAF bZIP Transcription Factor G
*cat*	Catalase
*tnfα*	Tumor Necrosis Factor α
*il1β*	Interleukin 1β
*tgfβ*	Transforming Growth Factor β1
*il10*	Interleukin 10
