# Supplementary material for: Dietary Fermented Mulberry Leaf Triggers Dose-Dependent Changes in Growth, Antioxidant Immune Status and Intestinal Microecology of Amur Sturgeon (Acipenser schrenckii)
Source: Animals (Basel). 2026 Sep 16;16(18):2918. doi: 10.3390/ani16182918 (PMC13603217; doi:10.3390/ani16182918)
Supplement: Supplementary file 1 [file animals-16-02918-s001.zip › animals-4524700-supplementary.pdf]

Supplementary Files

Table S1. Proximate nutrients and tannin concentrations of ML and FML (% dry matter).

Ingredient	Dry matter	Crude fat	Crude protein	Crude ash	Crude fiber	Tannin
ML[1]	99.93	4.46	17.01	12.30	9.44	21.96
FML[1]	99.93	4.12	18.98	16.17	9.86	12.73

1.ML, mulberry leaves; FML, fermented mulberry leaves.
All values are expressed as percentages of dry matter.

Table S2. Formulation and compositions of experimental diets (%).

Ingredients	Experimental diets			
	CK	FML2	FML4	FML6
Fish meal[1]	55.00	54.45	53.35	52.25
FML	0.00	2.00	4.00	6.00
Wheat middling	18.00	15.67	10.35	5.00
Wheat gluten meal	19.00	19.00	22.00	25.00
Fish oil[1]	3.50	3.50	4.00	4.30
Soybean oil[1]	3.00	3.00	3.00	3.00
L-Lysine[2]	0.05	0.50	1.00	1.50
Yeast extract	1.00	1.00	1.00	1.00
DL-Met[2]	0.00	0.20	0.40	0.80
$Ca(H_2PO_4)_2$	0.05	0.08	0.10	0.15
Thr[2]	0.00	0.20	0.40	0.60
Mineral premix[3]	0.20	0.20	0.20	0.20
Vitamin premix[4]	0.20	0.20	0.20	0.20
Total	100	100	100	100
Analyzed nutrient composition (%, in dry matter basis)				
Dry matter	95.3	94.7	95.2	95.7
Organic matter	88.3	88.5	87.6	87.4
Crude protein	46.5	46.7	46.6	46.7
Crude lipid	11.4	11.4	11.4	11.6
Gross energy (MJ/kg)	19.7	19.6	19.6	20.0

[1] Fish meal was supplied by Pesquera Centinela Co., Lima, Peru. Fish oil and Soybean oil were supplied by Han Ye Biotechnology Co.,Ltd. Shangdong, China.

[2] Referencing the ideal AA model of white sturgeo, calculated the supplementation level of CAA.

[3] Mineral premixes (mg/kg diet): $CuSO_4 \cdot 5H_2O$ 10; $FeSO_4 \cdot H_2O$ 300; $ZnSO4 \cdot H_2O$ 200; $MnSO_4 \cdot H_2O$ 80; KI (1%) 1.5; Na_2SeO_3 (10% Se) 10; $CoCl_2 \cdot 6H_2O$ (10% Co) 20; Zeolite 378.5.

[4] Vitamin premix (mg/kg diet): Vitamin A 20; Vitamin B1 12; Vitamin B2 10; Vitamin B6 15; Vitamin B12 (1%) 8; Niacinamide 100; Ascorbyl calcium phosphate (35%) 1000; Calcium pantothenate 40; Biotin (2%) 2; Folic acid 10; Vitamin E (50%) 400; Vitamin K3 20; Vitamin D3 10; Inositol 200; Choline chloride (50%) 4000; Corn protein powder 150.

Table S3. Amino acid composition of experimental diets (g/100 g crude protein).

Amino acid	Groups			
	CK	FML2	FML4	FML6
Essential amino acid, EAA				
Arginine	5.17	5.26	4.96	4.91
Histidine	3.11	3.16	3.11	3.15
Isoleucine	3.52	3.65	3.37	3.34
Lysine	6.46	7.09	7.25	7.55
Leucine	6.30	6.48	6.05	5.98
Methionine	2.27	2.58	2.79	3.46
Phenylalanine	3.56	3.64	3.35	3.29
Threonine	3.72	4.20	4.32	4.71
Tryptophan	0.83	0.81	0.81	0.76
Valine	4.19	4.33	4.05	4.03
Conditionally essential amino acid, CEAA				
Cystine	1.20	1.18	1.14	1.11
Glycine	5.19	5.27	5.16	5.19
Proline	3.88	3.84	3.66	3.63
Nonessential amino acid, NEAA				
Alanine	5.40	5.50	5.25	5.23
Aspartic acid	7.66	7.84	7.37	7.34
Glutamic acid	12.73	12.72	11.99	11.88
Serine	3.61	3.64	3.42	3.39

Table S4. Primer sequences for real-time PCR.

Gene	Sequence 5′-3′	Target size (bp)	Tm (°C)	Efficiency values (%)
β-actin	F: CGGTTTCGCTGGAGATGATG<br>R: TCAGTGAGCAGGACGGGGTG	135	60	102
tnfα	F: AAAATGTCCAGCCATCAAAGCA<br>R: GTGAGGAATGATCCCGTTGGT	132	60	93
il1β	F: TGGTGGAGAAGAGAACGCAAGATG<br>R: GCGTCACTGCCTGTCTCATGG	114	60	105
tgfβ	F: TGCTCCTGTGCTCGGCTCTC<br>R: CAGCTCCATGTCCAGAGTCTTGC	80	60	109

il10	F: GCAAAGCTGAGGGAGCTGAG<br>R: CCCAGATTGACAGCTGGAGG	194	60	107
keap1	F: CCAGACCGGATAACGAGCTGAATC<br>R: GCCTGGCTTGTGAGACTCTGTATC	101	60	93
nrf2	F: TGGAAGTGTCCAGCCTGAGT<br>R: GTCGGCAGTCCCTTCTTTGG	138	60	101
mafg	F: ACAGGCGCCAGGTCTTAGTA<br>R: GAACTCAACCACTGCCACAA	155	58	95
mafk	F: AAATCAGCCGACCACAAGAC<br>R: ACTGGATGGACTGGCAAGAG	161	58	103
cat	F: TGGACACCGACACATGAATGGC<br>R: GGCGATGGCGTTGTACAGGTC	190	60	109

Tm, melting temperature; F, forward primer; R, reverse primer.
tnfα = Tumor Necrosis Factor α; *il1β* = Interleukin 1β; *tgfβ*= Transforming Growth Factor β; *il10* = Interleukin 10; *keap1* = Kelch-like ECH-associated protein 1; *nrf2* = Nuclear Factor Erythroid 2-Related Factor 2; *mafg* = MAF BZIP Transcription Factor G; *mafk* = MAF bZIP Transcription Factor K; *cat* = Catalase.
